# Pathogenesis of Hemophagocytic Lymphohistiocytosis/Macrophage Activation Syndrome: A Case Report and Review of the Literature

**DOI:** 10.3390/ijms25115921

**Published:** 2024-05-29

**Authors:** Chiara Gioia, Marino Paroli, Raffaella Izzo, Lorenzo Di Sanzo, Elisabetta Rossi, Pasquale Pignatelli, Daniele Accapezzato

**Affiliations:** Division of Clinical Immunology, Department of Clinical, Anesthesiologic and Cardiovascular Sciences, Sapienza University of Rome, 00185 Rome, Italy; chiara.gioia@uniroma1.it (C.G.); marino.paroli@uniroma1.it (M.P.); raffaella.izzo@uniroma1.it (R.I.); lorenzo.disanzo@uniroma1.it (L.D.S.); e.rossi@policlinicoumberto1.it (E.R.); pasquale.pignatelli@uniroma1.it (P.P.)

**Keywords:** hemophagocytic lymphohistiocytosis, macrophage activation syndrome, T lymphoma

## Abstract

Hemophagocytic lymphohistiocytosis (HLH) is a life-threatening condition characterized by the uncontrolled activation of cytotoxic T lymphocytes, NK cells, and macrophages, resulting in an overproduction of pro-inflammatory cytokines. A primary and a secondary form are distinguished depending on whether or not it is associated with hematologic, infectious, or immune-mediated disease. Clinical manifestations include fever, splenomegaly, neurological changes, coagulopathy, hepatic dysfunction, cytopenia, hypertriglyceridemia, hyperferritinemia, and hemophagocytosis. In adults, therapy, although aggressive, is often unsuccessful. We report the case of a 41-year-old man with no apparent history of previous disease and an acute onset characterized by fever, fatigue, and weight loss. The man was from Burkina Faso and had made trips to his home country in the previous five months. On admission, leukopenia, thrombocytopenia, increased creatinine and transaminases, LDH, and CRP with a normal ESR were found. The patient also presented with hypertriglyceridemia and hyperferritinemia. An infectious or autoimmune etiology was ruled out. A total body CT scan showed bilateral pleural effusion and hilar mesenterial, abdominal, and paratracheal lymphadenopathy. Lymphoproliferative disease with HLH complication was therefore suspected. High doses of glucocorticoids were then administered. A cytologic analysis of the pleural effusion showed anaplastic lymphoma cells and bone marrow aspirate showed hemophagocytosis. An Epstein–Barr Virus (EBV) DNA load of more than 90000 copies/mL was found. Bone marrow biopsy showed a marrow localization of peripheral T lymphoma. The course was rapidly progressive until the patient died. HLH is a rare but usually fatal complication in adults of hematologic, autoimmune, and malignant diseases. Very early diagnosis and treatment are critical but not always sufficient to save patients.

## 1. Introduction

Hemophagocytic lymphohistiocytosis (HLH) is a potentially life-threatening syndrome characterized by the persistent activation of cytotoxic T lymphocytes and natural killer (NK) cells. The uncontrolled immune response is responsible for the production of pro-inflammatory cytokines and subsequent activation of macrophages, resulting in systemic inflammation. Hyperferritinemia is generally exploited as a biomarker, especially for the early identification of patients with a severe form of the disease. HLH is usually classified into primary or familial and secondary or reactive. Familial HLH (F-HLH) is typical in children and is caused by the presence of several genetic defects characterized by mutations or genetic variants that modulate cytolytic functions, lymphocyte survival, and inflammasome activation. Secondary or acquired HLH, which accounts for about 40 percent of total HLH cases, can result from the presence of neoplastic, infectious, or autoimmune disease. When HLH occurs in the context of a rheumatologic disease such as systemic juvenile idiopathic arthritis (sJIA), adult Still’s disease (ASD), or systemic lupus erythematosus (SLE), it is often referred to as macrophage activation syndrome (MAS) [1,2].

The pathophysiology of HLH is thought to be related to the inability of NK cells and CD8+ T cells to eliminate activated or infected cells, due to the lack of perforin-dependent granule-mediated cytotoxicity. This leads to the prolonged activation of NK cells, CD8+ T cells, and macrophages, resulting in excessive cytokine secretion or “cytokine storm”. Cytokine storm syndrome (CSS) encompasses a broad spectrum of similar but not identical systemic hyperinflammatory states characterized by elevated levels of circulating cytokines and the hyperactivation of immune cells. These can be triggered by pathogens, including SARS-CoV-2, neoplastic diseases, and autoimmune and autoinflammatory conditions [3,4]. In contrast to primary HLH, distinctive patterns of T lymphocyte activation and differentiation have been observed in patients with secondary HLH. An important role in the pathogenesis of HLH appears to be played by Epstein–Barr virus (EBV), which is the most commonly identified pathogen in both primary and secondary HLH. It has been hypothesized that EBV, by infecting B lymphocytes, may cause HLH through the secondary induction of CD8+ cytotoxic T lymphocytes [5]. A spectrum of many underlying immune dysfunctions is believed to predispose patients with systemic autoimmune disorders to the development of HLH. For example, it has been reported that some patients may have acquired functional abnormalities of perforin-mediated cytolysis by NK cells [6]. 

Clinically, HLH presents with fever, cytopenia, lymphadenopathy, hepatomegaly and splenomegaly, hepatic dysfunction, coagulopathy, hypertriglyceridemia, neurological dysfunction, and multiorgan failure. Laboratory data commonly consist of increased acute-phase reactants. However, both clinical manifestations and data obtained from laboratory investigations are often nonspecific. Therefore, diagnosis is a challenge for the physician. As a first step, in the presence of elevated ferritin levels, the rapid exclusion of various possible causes, including hematologic, infectious, and hepatic diseases, is essential. The rapid recognition of secondary HLH is critical to start treatment as quickly as possible [7]. A hallmark of HLH is the presence of hemophagocytosis in the bone marrow and lymphatic system [8]. Diagnostic scoring systems such as HScore and HLH-2004 criteria are commonly used for diagnosis [9,10]. Treatment is directed at reducing the extent of the immune response with immunosuppressive or myelosuppressive therapies, which can sometimes even complicate the condition of patients with multiorgan failure at the time of diagnosis. In addition, the search for possible triggers and their appropriate management is crucial.

## 2. Results

In January 2023, a 41-year-old man was admitted to the emergency department. He presented with high fever, fatigue, and reported a weight loss of 20 kg in the past 2 weeks. The patient, who apparently had no major pathologies in his medical history, was originally from Burkina Faso but had been living in Italy for about 7 years. He reported that he had made two trips to his home country, the last of which was within the previous 5 months. His condition immediately appeared very serious. Physical examination revealed axillary lymphadenopathy and decreased strength and sensation in the lower extremities, which had appeared in the previous 3 months. Laboratory investigations revealed anemia, leukopenia, thrombocytopenia, increased creatinine, aminotransferases, creatine phosphokinase (CPK), lactate dehydrogenase (LDH), and C-reactive protein (CRP). An abdominal ultrasound was performed, which showed pleural effusion and abdominal–pelvic effusion.

The patient was then admitted to the Department of Internal Medicine. He was alert and oriented. He presented with high fever, tachycardia, tachypnea, asthenia, and difficulty walking. The vital parameters were as follows: blood pressure 130/80 mmHg, pulse 110 beats per minute, respiratory rate 20 breaths per minute, and oxygen saturation 95% in room air. Supraclavicular, submandibular, cervical, axillary and inguinal lymphadenopathy were present. No skin eruptions or lesions were present, including the palms of the hands and soles of the feet. The heartbeat was rhythmic and no murmurs were detected. Hepatosplenomegaly was present. The patient presented with an occasional cough during the examination, with reduced lung sounds at the bases, especially on the right, and inspiratory crackles at the bases. There was no stiff neck, while significant lower limb weakness was present. Further blood tests revealed a prolonged international normalized ratio (INR) and activated partial thromboplastin time (aPTT). The blood level of CRP was 38.20 mg/dL, the erythrocyte sedimentation rate (ESR) normal and procalcitonin (PCT) significantly increased. Hyperferritinemia, hypertriglyceridemia, hypoalbuminemia and hypofibrinogenemia were also present. The results of all laboratory data are shown in Table 1.

**Figure 1:**
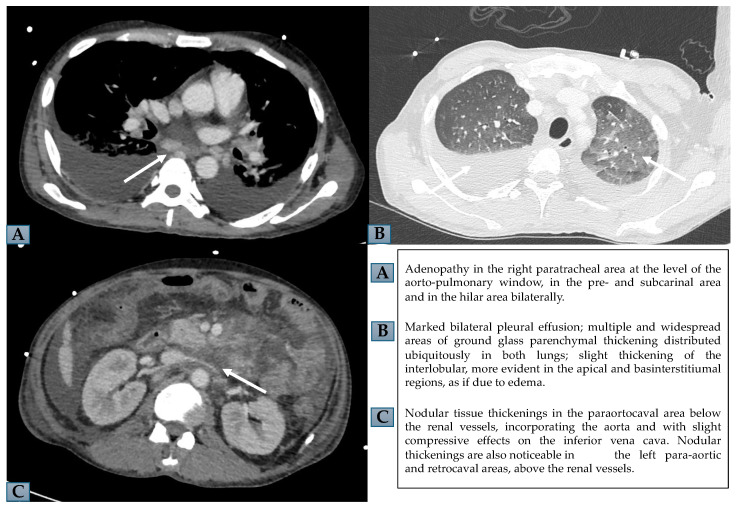
Chest and abdomen CT. (**A**,**B**) show mediastinal adenopathy and bilateral pleural effusion with ground glass parenchymal thickening, in chest CT. (**C**) displays nodular thickenings in paraortocaval and retrocaval areas, in abdomen CT.

On the basis of these findings, HLH was suspected and several diagnostic tests were promptly performed, considering all possible differential diagnoses with diseases associated with hyperferritinemia. First, also in view of the recent trip to the home country, we ruled out the infectious cause of the fever; the blood culture and urine culture, the rapid malaria test and thick-drop smear, and QuantiFERON-TB Gold (QFT) for the detection of the cell-mediated immune response to specific Mycobacterium tuberculosis antigens, human immunodeficiency virus (HIV) antibody test, hepatitis B virus (HBV) markers, hepatitis C virus (HCV) antibodies, EBV antibodies, and cytomegalovirus (CMV) antibodies were all negative. On the chest X-ray, an effusion in the right hemithorax and diffuse parenchymal thickening suggestive of pneumonia were detected. Autoimmunity testing, including antinuclear antibodies (ANA), extractable nuclear antigens (ENA) and antineutrophil cytoplasmic antibodies (ANCA), was also negative. On the suspicion of lymphoproliferative disease, a total body computed tomography (CT) scan was performed. CT showed bilateral pleural effusion, pulmonary thickening, peritoneal effusion, and mesenteric lymphadenopathy with hilar abdominal and paratracheal adenopathy (Figure 1). Antibiotic therapy was then started with vancomycin, piperacillin/tazobactam and azithromycin and a glucocorticoid (GC) pulse of methylprednisolone, followed by dexamethasone. 

During hospitalization, the patient’s condition progressively worsened with the appearance of an anasarcatic state and neurosensory changes leading to a comatose state. Brain magnetic resonance imaging (MRI) was then performed, which was negative. Dyspnea also worsened, so a thoracentesis was performed. The cytological analysis of the pleural effusion showed anaplastic lymphoma cells. EBV-DNA was 92474 gene copies/mL, while human herpesvirus-6 (HHV6) and human herpesvirus-8 (HHV8) DNA was not detected. 

**Figure 2:**
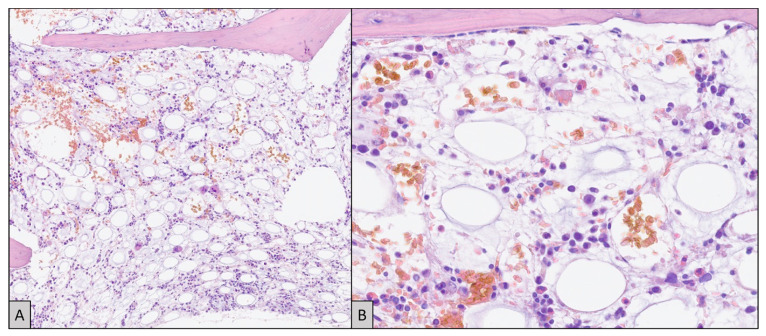
Bone marrow trephine biopsy. At low magnification (**A**) there is evident marrow hypoplasia, associated with atrophy of fat cells. At higher magnification (**B**) all hematopoietic lineages seem reduced, with marked dysmyelopoiesis and only relative sparing of some erythropoietic islands in the lower quadrants. No figures of hemophagocytosis could be recognized (Hematoxylin and eosin; original magnification: (**A**)—4×; (**B**)—20×).

Due to progressive multi-organ failure, the patient was transferred to the intensive care unit (ICU), where vital functions were supported and the diagnostic procedure continued. Bone marrow aspirate showed hypocellularity and hemophagocytosis, and the bone marrow biopsy confirmed a bone marrow localization of CD3+, CD8+, CD4−, CD5+, and CD20− peripheral T lymphoma (Figure 2). During hospitalization in the intensive care unit, the patient developed nosocomial Acinetobacter baumanii pneumonia and subsequent septic shock, which made it difficult to initiate anti-cytotoxic and immunosuppressive therapy. Thus, the final diagnosis was T-cell lymphoproliferative disease associated with hemophagocytic lymphohistiocytosis. HLH-2004 criteria were met with a high H-score of 303 points. The course was rapidly progressive over 6 days until the patient died.

## 3. Discussion

We report an illustrative case of HLH related to EBV-infection and hematologic malignancies. HLH and MAS are life-threatening systemic hyperinflammatory syndromes characterized by fever, elevated ferritin, cytopenia, hepatitis, disseminated intravascular coagulopathy, and central nervous system inflammation. These conditions are characterized by a high risk of progression to multiple organ failure, shock, and often have fatal outcomes [11]. Over the years, especially since the COVID pandemic, this condition has been variously referred to as HLH, MAS, CSS, “hyperinflammation”, “hyperferritinemic sepsis-induced multiorgan dysfunction”, or “SARS-CoV-2-associated multisystem inflammatory syndrome in children or adults” [12]. In 2022, the European Alliance of Association for Rheumatology (EULAR) defined systemic hyperinflammation as a state of excessive immune activation that could lead to HLH/MAS. In addition, three categories of factors contributing to the development of HLH/MAS have been defined: (1) genetic causes, (2) predisposing conditions such as sJIA, lymphoma, and some metabolic diseases, and (3) triggering factors such as infections and immunotherapy [13].

According to current knowledge, the first step in the pathogenesis of MAS would be a defect in the cytolytic activity of lymphocytes. Normally, cytotoxic cells are able to cause the apoptosis of hyperactivated cells, including macrophages and activated T cells. Such cells could then control the extent of the inflammatory response. A defect in this function may result in the overstimulation of the immune system, leading to multi-organ failure such as that observed in HLH. In addition, pro-inflammatory cytokines in the microenvironment, particularly interleukin (IL)-6, have been shown to reduce the cytolytic function of NK cells. The inability of NK cells and CD8+ T lymphocytes to lyse activated antigen presenting cells (APCs) induces the amplification of the downstream pro-inflammatory cytokine cascade. The “cytokine storm” in turn causes macrophage activation, contributing to multiorgan dysfunction. Several cytokines, including tumor necrosis factor (TNF), interferon (IFN)-γ and numerous interleukins such as IL-1, IL-6, IL-18 have been implicated in this process. In addition, genetic mutations in cytolytic pathway genes associated with fHLH, such as PRF1 and UNC13D, have been identified in a large subgroup of patients with MAS. These mutations are responsible for defects in the synthesis of proteins responsible for the production and transport of cytotoxic granules, leading to the apoptosis of target cells [14]. The role of macrophages in MAS has been widely recognized as these cells may be responsible for hemophagocytosis. These cells, on the other hand, also play a critical role in the regulation of the excessive immune response [15] due to their functional plasticity in response to various stimuli in the inflammatory microenvironment [16]. It has been suggested that the pro-inflammatory M1 and anti-inflammatory M2 phenotypes of macrophages may be two ends of a continuous spectrum of various intermediate phenotypes, finely regulated in response to external stimuli [17,18]. The degree of macrophage activation in MAS may thus reflect the heterogeneity of these cells in the inflammatory environment. Hemophagocytosis occurs in more advanced stages and is found in approximately 60% of biopsies of patients with HLH and MAS [19]. It has been shown that during disease progression, macrophages can switch from a pro-inflammatory to an anti-inflammatory phenotype, thus attempting to suppress the extremely hyperactive inflammatory state in patients with fulminant disease. Similarly, ferritin is considered cytoprotective because of its ability to sequester free Fe^2+^, reducing endothelial apoptosis mediated by increased oxidative stress [20]. Together with the anti-inflammatory role of IL-10, these features suggest that the increase in the number of hemophagocytic CD163+ macrophages and ferritin in MAS could therefore be a compensatory mechanism rather than a cause of pathology in MAS.

Because HLH is a hyperinflammatory syndrome, increased serum ferritin levels can be exploited as a very useful diagnostic marker, and hyperferritinemia is included among the clinical diagnostic criteria for HLH. Nevertheless, it has been found that HLH is not diagnosed in most critically ill adult patients [21], in part because of the high degree of difficulty in interpreting elevated ferritin levels in the differential diagnosis. Indeed, hyperferritinemia is associated with a wide range of conditions. However, three causes explain more than two thirds of cases of hyperferritinemia ≥ 5000 μg/L, such as infectious diseases, HLH and acute hepatitis. In adult critically ill patients without HLH, sepsis or septic shock, liver disease and hematologic neoplasms are thus the main factors associated with hyperferritinemia. 

Hyperferritinemia in liver disease may result from damaged liver cells [22], particularly hepatocytes, which contain high amounts of iron and synthesize ferritin [23]. Hyperferritinemia in sepsis is related to a pro-inflammatory state, with increases in IL-6, IL-18, IFNγ, and sCD163, a decrease in the IL-10/TNFα ratio, and the production of elevated levels of reactive oxygen species (ROS) [24,25], leading to the expression of ferritin as an antioxidative stress response and as an acute-phase reactant [26]. Among hematologic diseases, T/NK cell lymphomas (NKTL) have been shown to be characterized by the highest serum ferritin levels. Importantly, ferritin values generally correlate with the level of inflammation in neoplastic diseases and proliferation rates in T/NK cell lymphomas [27,28]. Therefore, ferritin may be an indicator of disease severity in hematologic malignancies. Based on these considerations, HLH-2004 criteria should be applied in hyperferritemic patients to exclude HLH, especially in the context of liver disease, hematologic neoplasms, sepsis, or septic shock [9]. On the other hand, hematologic neoplasms may not only be the cause of hyperferritinemia but also the trigger in patients with HLH [29]. The HLH-2004 diagnostic criteria have been shown to differentiate between HLH and non-HLH as causes of hyperferritinemia in critically ill adult patients with good sensitivity and specificity [30]. Our patient presented a very high value of ferritinemia and because of his clinical presentation and other laboratory tests, we immediately applied HLH-2004 diagnostic criteria. Fever, cytopenia, splenomegaly, hypertriglyceridemia and/or hypofibrinogenemia, ferritin ≥500 μg/L and hemophagocytosis allowed five out of seven criteria to be met; an H-score of 303 points was equivalent to a very high probability (99%) of hemophagocytic syndrome (optimal cutoff is 169). Once the diagnosis of HLH was confirmed, it was crucial to recognize the triggers to provide adequate therapy as early as possible. EBV infection and EBV+ lymphoma were the coexistent triggers in our patient.

Neoplasm-associated HLH (M-HLH) is a rare type of HLH described in about 1% of adults with hematologic malignancies [31] and characterizes 40–70% of all HLH cases in adults [32]. M-HLH is most commonly found in association with lymphomas, with an incidence rate of about 2.8%. HLH-associated lymphomas are predominantly T-cell lymphomas, followed by diffuse large B-cell lymphoma and Hodgkin’s lymphoma [33,34]. The distribution of the etiologies of M-HLH varies by geographic region. For example, EBV-driven lymphoproliferative disorders are more common in East Asia [35]. Probably, in the case of lymphomas, the cytokine storm is related to persistent antigen stimulation and the hypersecretion of proinflammatory cytokines by neoplastic cells [36]. The diagnosis of M-HLH can be challenging because many of the markers listed in the HLH-04 criteria (e.g., fever, splenomegaly, cytopenia) may be abnormal in patients with hematologic malignancies. On the other hand, the presence of hemophagocytosis on bone marrow biopsy is neither a sensitive nor specific marker of HLH [37]. In one study, only 17% of adult patients with ferritin above 50,000 ng/mL had HLH, while 32% had a hematologic malignancy [38]. The prognosis of refractory M-HLH is inauspicious, and treatment options are mostly borrowed from studies of nonmalignant HLH. Ruxolitinib, a Janus kinase 1/2 (JAK1/2) inhibitor, has been shown to be effective in some forms of secondary HLH both as monotherapy and in combination with doxorubicin, etoposide and dexamethasone [39,40]. The efficacy of anti-cytokine therapy targeted with biologic drugs to IL-1, IL-6, IL-18 and IFN-γ in M-HLH is still unknown. However, blocking IL-1 with the IL-1 receptor antagonist, anakinra, has been shown to be useful in treating some patients with M-HLH [41]. In cases of refractory M-HLH that can achieve partial or complete response, allogeneic HSCT is strongly recommended.

Referring to the diagnosis of the patient under this study, he presented with a bone marrow localization of peripheral T-cell lymphoma (PTCL), classified as primary EBV-positive T-cell and NK-cell nodal lymphoma, a rare disease, classified in 2016 by the WHO as a variant of PTCL [42]. This form of lymphoma occurs most commonly in elderly and/or immunodeficient patients. This lymphoma lacks nasal involvement and is more often T-line rather than NK-line [43]. EBV+ nodal CTL, on the other hand, can develop in a wide range of ages, from infants to the elderly, but predominantly affects middle-aged and elderly people, with a reported median age of 61–64 years. Epidemiologically, a male predominance has been described, with an M:F ratio of 1.5–3.8:1. Some cases may be associated with autoimmune diseases and/or immunosuppressive treatment [44]. Lymph nodes typically show a monomorphic infiltration of atypical cells, in which tumor cells express CD3 and CD2 and lack CD5 and CD4. CD56 is positive in <20% of cases [45]. The median overall survival of EBV+ nodal disease is 2.5–8.0 months, significantly lower than that of NKTL (26–50 months) or PTCL (16–20 months) [46].

The treatment of patients with suspected HLH/MAS requires a careful evaluation of the risk–benefit ratio [47]. From the data in the literature, intensive care unit admission is necessary in about half of adults with HLH/MAS [48], requiring mechanical ventilation, vasopressor/inotropic therapy, and renal replacement therapy [49]. Because immunomodulatory treatment has dramatically improved survival in most patients with HLH/MAS with features at a high risk of progression [13], empiric immunomodulation in the early stages, such as the use of glucocorticoids (GCs), the recombinant IL-1 receptor antagonist (IL-1RA) anakinra, and/or the administration of intravenous immunoglobulin (IVIg), is strongly recommended [13]. Regarding GC therapy, “pulsed” doses of intravenous methylprednisolone are effective in forms associated with severe rheumatic and neuroinflammatory diseases [50], while dexamethasone is used because of its good penetration into the central nervous system. In general, short-acting GCs may be preferred in rapidly changing diagnostic scenarios. As observed in the literature, we immediately started pulse doses of IV methylprednisolone, requiring an intensive care unit admission. Anakinra appears to be a generally safe and effective treatment for many autoinflammatory and rheumatic disorders and, because of its rapid onset and short half-life, is preferred in rapidly evolving patients [51]. B-cell depletion may be useful in some patients with EBV-HLH [52]. The early initiation of chemotherapy-based treatment regimens with etoposide has been shown to be lifesaving for patients with primary HLH and severe EBV-HLH [47]. Etoposide is not indicated for most non-EBV infections [53] and its efficacy in neoplasm-associated HLH is currently unclear. For patients with increased inflammation and/or worsening organ damage despite early immunomodulation, the escalation of treatment with higher doses of GC and/or alternative agents such as cyclosporine, rituximab, ruxolitinb, and emapalumab should be considered. Our patients presented a rapidly progressive evolution with the inability to set up a targeted immunosuppressive therapy, once triggers were identified. Increasing evidence supports the involvement of the IFNγ pathway in HLH/MAS. The IFNγ-neutralizing antibody, emapalumab, was recently approved in the United States for the treatment of refractory, recurrent, or progressive HLH [54]. Ruxolitinib and some JAK inhibitors target the activity or synthesis of several cytokines, including IFNγ, and have shown promising initial results in HLH/MAS [55]. To this end, ongoing clinical trials to test the safety and efficacy of agents such as ruxolitinib (NCT04551131), alemtuzumab (NCT02472054), tadekinig alfa (NCT03113760), emapalumab (NCT05001737), and MAS825 (NCT04641442) in different HLH/MAS settings are crucial.

## 4. Materials and Methods

The medical history was collected for the previous five years of hospitalization. Clinical and laboratory data were collected from the original electronic medical record of our hospital. Hematologic and histopathologic investigations, including conventional microscopy and immunohistochemistry, were performed by the Department of Radiologic, Oncologic and Anatomopathological Sciences and the Department of Hematology of Policlinico Umberto I in Rome. The final diagnosis of HLH was made according to the criteria defined by the HLH Study Group of the Histiocyte Society [9]. A literature review was conducted: publications (reviews, original articles and case report series) from the period 2004–2024 were analyzed. Research parameters were: “HLH”, “MAS”, “CSS” and “T lymphoma”.

## 5. Conclusions

HLH or MAS are life-threatening systemic hyperinflammatory syndromes with a high risk of progression to multiple organ failure, shock, and often death. Elevated serum ferritin levels are an important diagnostic and prognostic index. Very early diagnosis with the underlying cause and the timely initiation of immunosuppressive therapy is essential.

## Figures and Tables

**Table 1:** Laboratory data.

Variable	In Medicine Department	Reference Range
Creatinine (mg/dL)	1.60	0.70–1.20
Na^+^ (mmol/L)	124	136–145
K^+^ (mmol/L)	4.5	3.4–5.5
Ca^++^ (mmol/L)	1.70	2.10–2.50
Proteins (g/dL)	4.2	6.0–8.2
Albumin (g/dL)	1.9	3.5–5.5
Amylase (U/L)	141	28–100
Lipase (U/L)	102	13–60
AST (U/L)	508	9–45
ALT (U/L)	148	10–40
γGT (U/L)	130	8–61
CPK (U/L)	1544	20–200
LDH (U/L)	2049	135–225
Triglycerides (mg/dL)	391	45–236
Ferritin (µg/L)	>8000	30–400
CRP (mg/dL)	3.82	0–0.5
ESR (mm/h)	5	0–25
PCT (ng/mL)	11.56	0.02–0.064
Hb (g/dL)	11.1	13.5–16.5
WBC (×10^9^/L)	3.16	4.40–11.30
N (×10^9^/L)	2.64	1.80–7.70
L (×10^9^/L)	0.30	1.80–4.80
PLT (×10^9^/L)	60	150–450
Fibrinogen (mg/dL)	84	150–400
INR	1.30	0.81–1.20
aPTT ratio	1.31	0.8–1.30

Na^+^—Sodium; K^+^—Potassium; Ca^++^—Calcium; AST—aspartate aminotransferase; ALT—alanine aminotransferase; γGT—gamma glutamyl transpeptidase; CPK—creatine phosphokinase; LDH—lactate dehydrogenase; CRP—C-Reactive Protein; ESR—erythrocyte sedimentation rate; PCT—procalcitonin; Hb—hemoglobin; WBC—white blood cells; N—neutrophils; L—lymphocytes; PLT—platelets; INR—international normalized ratio; aPTT—ratio Activated Partial Thromboplastin Time.

## Data Availability

The data used in this study are available from the corresponding author upon request.
